# Supplementary figures and images for: Long term outcomes of patients with tuberculous meningitis: The impact of drug resistance
Source: PLoS One. 2022 Jun 24;17(6):e0270201. doi: 10.1371/journal.pone.0270201 (PMC9232145; doi:10.1371/journal.pone.0270201)

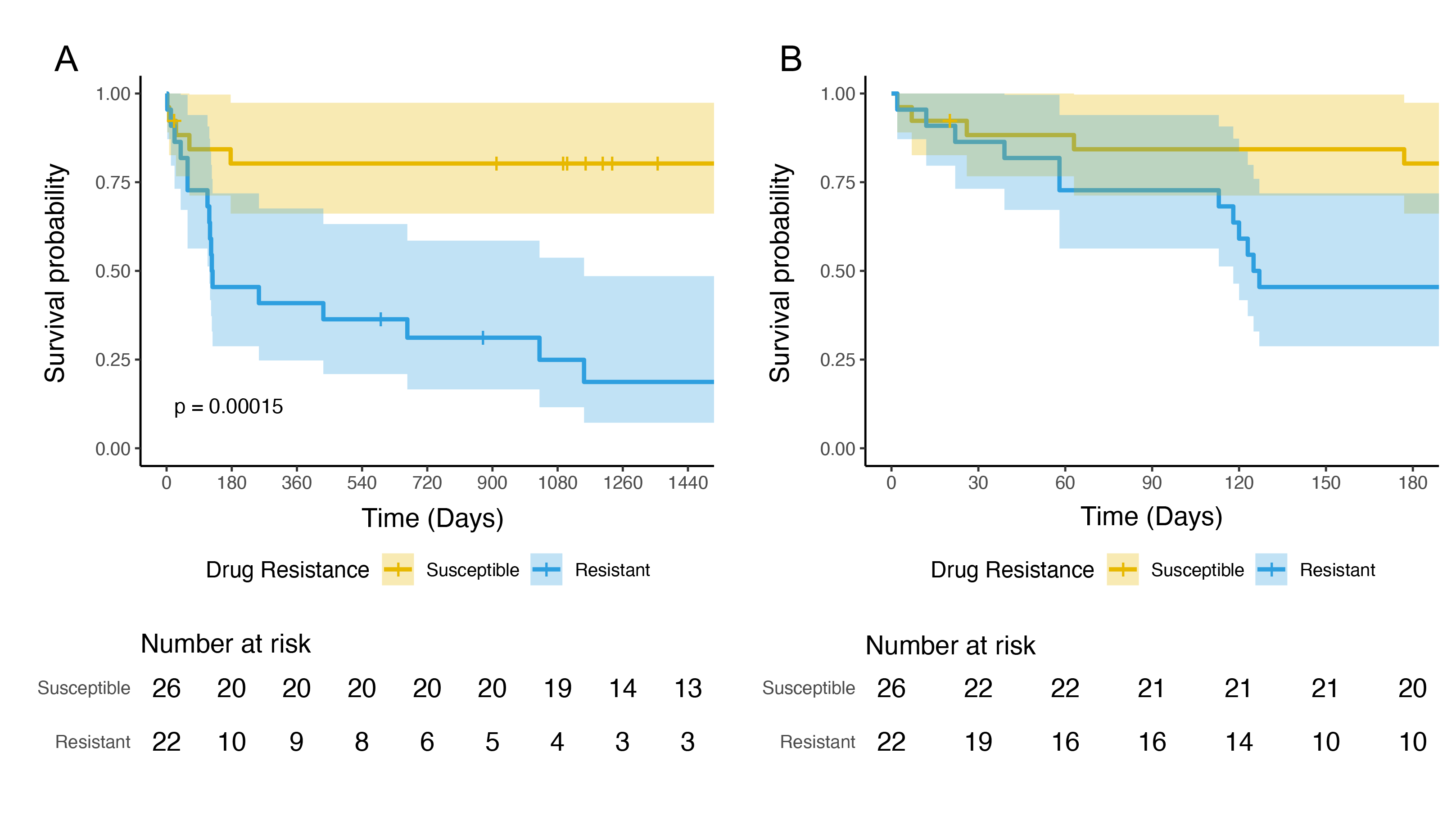

Supplement: S1 Fig — Kaplan Meier survival curves for (A) all patients with microbiologically confirmed tuberculosis meningitis (TBM; n = 48) over the full period of observation and (B) over first 6 months. Mortality in persons with drug susceptible-TBM is depicted by the yellow line and in persons with drug resistant-TBM by the blue line. The shaded region surrounding the line depicts the 95% confidence interval of the mortality estimate. (TIF) [file pone.0270201.s001.tif]
