# Supplementary material for: Long term outcomes of patients with tuberculous meningitis: The impact of drug resistance
Source: PLoS One. 2022 Jun 24;17(6):e0270201. doi: 10.1371/journal.pone.0270201 (PMC9232145; doi:10.1371/journal.pone.0270201)
Supplement: S1 Table — Abbreviations used: HIV, human immunodeficiency virus; ART, anti-retroviral therapy; TB, tuberculosis; TBM, tuberculous meningitis; CNS, central nervous system; MRI, magnetic resonance imaging; CT, computed tomography; CXR, chest X-ray; LTFU, lost to follow up; ATT, anti-tuberculosis therapy; CSF, cerebrospinal fluid; WCC, white cell count. Note: Three patients excluded for whom final outcome was not determined. 1. Defined by the Uniform Tuberculous Meningitis Research Case Definition Criteria. (DOCX) [file pone.0270201.s002.docx]

**S1 Table. Characteristics of patients with tuberculosis meningitis by vital status.**

| **Characteristic** | **Alive**  n = 161 (%) | **Dead**  n = 73 (%) |  |
| --- | --- | --- | --- |
| Mean age, years (SD) | 40 (15) | 53 (16) |  |
| Male sex | 95 (59) | 46 (63) |  |
| Weight < 50 kilograms | 6 (4) | 4 (6) |  |
| History of imprisonment | 11 (7) | 13 (18) |  |
| Contact to active tuberculosis case | 18 (11) | 7 (10) |  |
| **HIV** | | |  |
| HIV infected | 14 (9) | 20 (27) |  |
| New diagnosis HIV | 7 (4) | 5 (7) |  |
| CD4, cells/ul, median (IQR) | 79 (36, 166) | 31 (20, 58) |  |
| ART experienced | 6 (4) | 13 (18) |  |
| **Co-morbidities** | | |  |
| Hepatitis C viral antibody positive | 11 (7) | 20 (27) |  |
| History of intravenous drug use | 7 (4) | 10 (14) |  |
| Alcohol Use Disorder | 5 (3) | 4 (6) |  |
| Diabetes | 7 (4) | 5 (7) |  |
| **Case Definition** | | |  |
| New Case | 146 (91) | 49 (67) |  |
| Relapse | 7 (4) | 4 (6) |  |
| Treatment after LTFU | 2 (1) | 9 (12) |  |
| Treatment after failure | 1 (1) | 4 (6) |  |
| Other | 5 (3) | 7 (10) |  |
| **Presentation** | | |  |
| Days since first neurologic symptom, median (IQR) | 10 (7, 16) | 10 (7, 14) |  |
| TBM Grade 1 | 30 (19) | 3 (4) |  |
| TBM Grade 2 | 106 (66) | 34 (47) |  |
| TBM Grade 3 | 25 (16) | 35 (49) |  |
| Altered Mental Status | 125 (78) | 67 (92) |  |
| Fever | 150 (93) | 61 (84) |  |
| Headache | 152 (94) | 62 (85) |  |
| Vomiting | 113 (70) | 32 (44) |  |
| Nuchal rigidity | 149 (93) | 67 (92) |  |
| Seizures | 18 (11) | 5 (7) |  |
| Cranial nerve palsy | 41 (25) | 22 (30) |  |
| Urinary retention | 30 (19) | 21 (29) |  |
| Hemiplegia | 8 (5) | 9 (12) |  |
| Paraplegia | 13 (8) | 5 (7) |  |
| **Site of Disease** |  |  |  |
| Evidence of extra-CNS disease | 33 (20) | 37 (51) |  |
| Pulmonary disease | 24 (15) | 36 (50) |  |
| **Care prior to admission** | | |  |
| Received other antibiotic therapy prior to admission | 60 (39) | 27 (45) |  |
| **Baseline CSF data** | | |  |
| CSF WCC, cells/ul, mean (SD) | 231 (293) | 170 (169) |  |
| CSF protein, mg/dl, mean (SD) | 153 (209) | 192 (275) |  |
| CSF glucose, mg/dl, mean (SD) | 46 (25) | 34 (18) |  |
| **Microbiology** | | |  |
| CSF Xpert positive | 14 (9) | 14 (19) |  |
| CSF culture positive | 20 (12) | 17 (23) |  |
| Any *Mtb* confirmation in CSF sample | 24 (15) | 22 (30) |  |
| Non-CSF sample smear positive | 8 (5) | 16 (22) |  |
| Non-CSF sample Xpert positive | 32 (20) | 36 (49) |  |
| Non-CSF sample culture positive | 26 (16) | 30 (41) |  |
| Any *Mtb* confirmation in non-CSF sample | 35 (22) | 37 (51) |  |
| Any *Mtb* confirmation in CSF or non-CSF sample | 48 (30) | 41 (56) |  |
| **Classified as drug-resistant** | 11 (7) | 24 (33) |  |
| **Imaging** | | |  |
| Either MRI brain or CT head performed | 113 (70) | 52 (71) |  |
| Any CNS imaging abnormality | 86 (53) | 48 (66) |  |
| **Mean Baseline labs** | | |  |
| Hemoglobin (SD) | 12.6 (1.9) | 11.7 (2.2) |  |
| Sodium (SD) | 131 (8) | 131 (9) |  |
| Creatinine (SD) | 77 (22) | 83 (32) |  |
| Albumin (SD) | 35 (7) | 32 (6) |  |
| **Case definition^1^** |  |  |  |
| Definite TBM | 25 (16) | 22 (30) |  |
| Probable | 19 (12) | 12 (16) |  |
| Possible | 112 (70) | 39 (53) |  |
| Unlikely | 5 (3) | 0 |  |
| **Follow-up time, days (Median, IQR)** | 1595 (1150-1864) | 125 (21-1153) |  |
| Abbreviations used: HIV, human immunodeficiency virus; ART, anti-retroviral therapy; TB, tuberculosis; TBM, tuberculous meningitis; CNS, central nervous system; MRI, magnetic resonance imaging; CT, computed tomography; CXR, chest X-ray; LTFU, lost to follow up; ATT, anti-tuberculosis therapy; CSF, cerebrospinal fluid; WCC, white cell count  Note: Three patients excluded for whom final outcome was not determined  1. Defined by the Uniform Tuberculous Meningitis Research Case Definition Criteria | | |  |
