# Supplementary material for: Long term outcomes of patients with tuberculous meningitis: The impact of drug resistance
Source: PLoS One. 2022 Jun 24;17(6):e0270201. doi: 10.1371/journal.pone.0270201 (PMC9232145; doi:10.1371/journal.pone.0270201)
Supplement: S2 Table — Abbreviations: DR, drug resistant; HIV, human immunodeficiency virus; TBM, tuberculous meningitis. (DOCX) [file pone.0270201.s003.docx]

**Supplemental Table 2**: **Multivariate analysis of predictors of mortality for patients with probable or definite tuberculosis meningitis**

| **Variables** | **Adjusted Hazards Ratio** | **95% CI** | **p-value** |
| --- | --- | --- | --- |
| Treated for DR TBM |  |  |  |
| < 90 days | 1.24 | 0.42, 3.65 | 0.7 |
| > 90 days | 9.2 | 2.86, 29.63 | <0.001 |
| HIV | 2.13 | 0.98, 4.67 | 0.06 |
| Sex | 1.39 | 0.59, 3.26 | 0.46 |
| Age | 1.04 | 1.01, 1.07 | 0.005 |
| Grade 3 TBM | 2.05 | 0.97, 4.34 | 0.06 |
| Abbreviations: DR, drug resistant; HIV, human immunodeficiency virus; TBM, tuberculous meningitis | | | |
